# Ocular and systemic immune profiles associated with cystoid macular edema in retinitis pigmentosa

**DOI:** 10.3389/fopht.2025.1653404

**Published:** 2025-09-05

**Authors:** Yan Tao, Huanyu Zhao, Sakurako Shimokawa, Masatoshi Fukushima, Kohta Fujiwara, Takahiro Hisai, Kaho Yamamoto, Ayako Okita, Koh-Hei Sonoda, Yusuke Murakami

**Affiliations:** 1 Department of Ophthalmology, Graduate School of Medical Sciences, Kyushu University, Fukuoka, Japan; 2 Department of Ophthalmology, Oita University, Oita, Japan

**Keywords:** retinitis pigmentosa, cystoid macular edema, neuroinflammation, cytokines, chemokines

## Abstract

**Purpose:**

We aimed to investigate the local and systemic inflammatory profiles associated with cystoid macular edema (CME) in patients with retinitis pigmentosa (RP).

**Patients and methods:**

Paired aqueous humor and serum samples were collected at the time of cataract surgery from 37 eyes of 37 patients with typical RP, including 29 without CME and eight with CME. The concentrations of cytokines and chemokines were determined using a multiplexed immunoassay (Q-Plex). Group comparisons were conducted to assess differences in the inflammatory molecule levels between the RP patients with and without CME. Correlations among the intraocular parameters, the systemic inflammatory molecules, and the CME status were analyzed.

**Results:**

Compared to RP patients without CME, those with CME showed significantly increased aqueous levels of interleukin 23 (IL-23) (*p* = 0.002), I-309 (*p* = 0.039), and growth-related oncogene alpha (GROα) (*p* = 0.042). A multiple-factor analysis further supported a potential association between CME formation and an IL-23-related inflammatory network characterized by aqueous IL-23, IL-8, GROα, eotaxin, I-309, serum IL-23, and IFN-γ.

**Conclusion:**

These findings suggest that both intraocular and systemic immune activation may play a role in the development of CME in patients with RP. Specifically, IL-23-driven inflammation may be associated with macular fluid accumulation. Further longitudinal studies in larger cohorts are necessary to elucidate these relationships and explore their clinical implications.

## 1 Introduction

Retinitis pigmentosa (RP) is the most common inherited retinal degenerative disease, affecting approximately 1 in 4,000 individuals globally (1, 2). RP is characterized by sequential rod and cone photoreceptor degeneration, which eventually leads to irreversible vision loss (2–5). Cystoid macular edema (CME) is a common complication in RP. It has been observed in 5%–49% of individuals with RP (3, 6) and is characterized by intraretinal fluid accumulation within cystic spaces of the macula, significantly impairing central vision and adversely affecting quality of life (7, 8). The precise pathophysiological mechanisms that underlie the development of CME in patients with RP have not been established.

Growing evidence indicates that the key contributors to the pathogenesis of RP are neuroinflammation and disruption of the blood–retinal barrier (BRB) (7). Numerous inflammatory mediators and lymphocytes have been shown to be elevated in the eyes of patients with RP (7, 9–13). Neuroinflammation promotes the development of retinal vascular permeability, which results in leukocyte infiltration and a self-amplifying cascade of cytokine and chemokine release (14). In an earlier study of our research group, it was revealed that the serum interleukin 8 (IL-8) and RANTES (regulated activation normal T-cell expressed and secreted) levels of patients with RP are significantly elevated, with IL-8 showing a negative correlation with patients’ central visual function (15). Moreover, a paired analysis revealed significant correlations between the aqueous and serum levels of IL-23, I-309, IL-8, and RANTES in a study of patients with RP, which showed that aqueous IL-23 was associated with faster visual acuity loss (10, 15). These findings suggest that RP involves peripheral immune activation and a close interplay between systemic and ocular inflammatory responses. However, the associations between these inflammatory changes in RP and the occurrence of CME have not been identified.

Building on this foundation, we conducted the present study to investigate the inflammatory profile associated with CME in patients with RP. We compared RP patients with and without CME based on their aqueous humor and serum inflammatory molecule levels and peripheral immune parameters. By identifying key inflammatory signatures linked to the occurrence of CME, we sought to obtain further insights into the immunopathological mechanisms underlying this common complication of RP.

## 2 Patients and methods

### 2.1 Patients

The study protocol was approved by the Institutional Review Board of Kyushu University Hospital (Fukuoka, Japan), and patient’s informed consent was obtained in accordance with the Declaration of Helsinki. Patients with ocular conditions other than RP, such as glaucoma, age-related macular degeneration (AMD), or uveitis, and those under anti-inflammatory treatment were excluded. We enrolled 37 eyes of 37 patients with typical RP (29 patients without CME and eight with CME) who underwent cataract surgery at Kyushu University Hospital during the period 2019–2023.

Aqueous humor samples were collected from each patient during the cataract surgery, and paired peripheral blood samples were obtained the same day. In cases in which both of the patient’s eyes were eligible, only the right eye and its corresponding serum sample were included for analysis.

The diagnosis of typical RP was established based on the patient’s clinical history, including night blindness and progressive peripheral visual field constriction or ring scotoma, along with hallmark findings, e.g., attenuated retinal vessels, bone spicule-like pigmentation in the mid-to-peripheral retina, and markedly diminished electroretinography (ERG) responses. In all eight of the RP patients with CME, the CME was clinically evident on a fundus examination and was confirmed by optical coherence tomography (OCT) prior to cataract extraction. Genetic inheritance patterns were inferred from identified mutations.

The patients’ baseline data were retrieved from their electronic medical records, including: age, sex, weight, body mass index (BMI), medication history, macular pathology, systemic comorbidities (i.e., hypertension, hyperlipidemia, diabetes mellitus, fatty liver disease, and autoimmune disorders), and lifestyle factors (tobacco smoking and alcohol consumption habits).

### 2.2 Clinical examination

Each patient’s best-corrected visual acuity (BCVA) was assessed using either a Landolt C decimal chart (CV-6000, Tomey, Nagano, Japan, or AVC-36, Kowa Pharmaceuticals, Tokyo, Japan) at a testing distance of 5 m or with single optotype cards (HP-1258, Handaya, Tokyo, Japan) when necessary. Acuity values were converted to logarithm of the minimum angle of resolution (logMAR) units for statistical analyses. The smallest Landolt ring correctly identified by a patient in ≥60% (in three or more out of five) of presentations was used as the patient’s BCVA value.

For all 37 patients, the following measurements were obtained as described (10, 15): the result of an automated static perimetry test by a Humphrey field analyzer (HFA) (Humphrey Instruments, San Leandro, CA, USA), the averaged retinal sensitivity at 4 or 12 central points, and the baseline spectral-domain (SD) OCT values (Cirrus, Carl-Zeiss Meditec, Dublin, CA, USA). All of the patients also underwent a comprehensive fundus evaluation with a multimodal retinal imaging system.

### 2.3 Measurements of cytokines and chemokines

Aqueous humor and serum samples were collected as described (10), and a Q-Plex™ Human Cytokine multiplex immunoassay (Quansys Biosciences, West Logan, UT, USA) was used to determine the concentrations of 15 cytokines [interleukin 1 alpha (IL-1α), IL-1β, IL-2, IL-4, IL-5, IL-6, IL-10, IL-12p70, IL-13, IL-15, IL-17, and IL-23; interferon gamma (IFN-γ), tumor necrosis factor alpha (TNF-α), and TNF-β] and nine chemokines [eotaxin, growth-related oncogene alpha (GROα), I-309, IL-8, interferon gamma-inducible protein 10 (IP-10), monocyte chemotactic protein 1 (MCP-1), MCP-2, RANTES (regulated activation normal T-cell expressed and secreted), and thymus and activated-regulated chemokine (TARC)]. Detailed assay protocols followed the manufacturer’s instructions and have been described (10).

### 2.4 Statistical analyses

Continuous variables such as age, BMI, BCVA, central foveal thickness (CFT), ellipsoid zone length (EZL), mean deviation (MD), and the four-point and 12-point central retinal sensitivities are expressed as the mean ± standard deviation (SD) and were compared using the Mann–Whitney test. Categorical variables including sex, smoking and alcohol consumption habits, systemic and ocular complications, and inheritance patterns are summarized as counts (percentages) and were analyzed using Fisher’s exact test. The cytokine, chemokine, and systemic blood parameters are summarized using the median and interquartile range (IQR) due to their non-normal distribution. Group comparisons between the RP patients with and without CME were performed using the Mann–Whitney test for the aqueous and serum molecule concentrations and the peripheral immune parameters, including the lymphocyte percentage (%LYMPH), the monocyte percentage (%MONO), the white blood cell count (WBC), the C-reactive protein (CRP), and the lymphocyte-to-neutrophil ratio (LNR). A two-tailed *p*-value <0.05 was considered significant.

### 2.5 Multiple-factor analysis

To explore the integrated structure of the inflammatory and clinical variables in the 37 RP patients with/without CME, we performed a multiple-factor analysis (MFA) using 40 continuous variables, including the cytokine/chemokine levels, the CFT, and the peripheral immune parameters. The inflammatory variables were grouped according to previously defined clusters based on the results of a hierarchical clustering analysis (10). These included six cytokine/chemokine groups: group A (aqueous RANTES, TARC, IP-10, MCP-1, and serum RANTES), group B (aqueous IL-6, serum MCP-1, and TARC), group C (aqueous IL-23, eotaxin, GROα, IL-8, I-309, serum IL-23, and IFN-γ), group D (serum eotaxin, GROα, I-309, IP-10, and MCP-2), group E (serum IL-10, IL-17, IL-2, IL-8, IL-4, IL-15, IL-6, IL-12, IL-1α, IL-13, and IL-5), and group F (aqueous MCP-2 and serum TNFα).

In addition, two new parameters were incorporated into the MFA: CFT and Peripheral_immune (five variables: %LYMPH, %MONO, WBC, CRP, and LNR). To distinguish between patients with and without CME, the MFA was conducted using the FactoMineR software package ver. 2.10 (Agrocampus Ouest, Rennes, France), and visualization was performed using the factoextra package ver. 1.0.7 (Kassambara, Marseille, France) in R.

## 3 Results

### 3.1 Baseline characteristics

**Table 1** summarizes the patients’ baseline clinical characteristics. The mean ages were 71 years (range, 48–81 years) in the eight RP patients with CME and 62 years (range, 41–81 years) in the 29 patients without CME. There was a statistically significant difference between groups (*p* = 0.032). No significant differences were observed between the groups in terms of sex distribution, visual acuity, BMI, tobacco or alcohol habits, CFT, EZL, MD, central four-point or 12-point retinal sensitivity, systemic diseases, or causative gene distributions.

**Table 1:** Baseline clinical characteristics of all patients.

Characteristic	RP		*p*-value
	Without CME	With CME	
Eyes (patients)	29 (29)	8 (8)	
Sex, women (%)	18 (62)	4 (50)	0.69^b^
Age (years) (range)	62.76 ± 10.36 (41–81)^a^	71.00 ± 10.39 (48–81)^a^	0.032^c^
VA, logMAR	0.43 ± 0.56^a^	0.35 ± 0.49^a^	0.814^c^
CFT	231.54 ± 10.26^a^	313.33 ± 35.05^a^	0.067^c^
EZL	2634.58 ± 574.42^a^	3051.17 ± 768.80^a^	0.586^c^
MD	−14.65 ± 1.96^a^	−16.25 ± 3.76^a^	0.982^c^
Four-point sensitivity	23.76 ± 1.76^a^	28.08 ± 2.91^a^	0.188^c^
Twelve-point sensitivity	22.25 ± 1.79^a^	24.99 ± 3.55^a^	0.388^c^
Smoking habit, *n* (%)	3 (10)	1 (13)	1^b^
Alcohol habit, *n* (%)	7 (24)	2 (25)	1^b^
Antihistamine, *n*	0	0	
Macular complications, *n*			<0.0001^b^
ERM	4	0	
CME	0	8	
VMTS	1	2	0.39^b^
Lamellar MH	1	0	
BMI (kg/m^2^) mean ± SD, *n* (%)	24.55 ± 3.78	23.26 ± 2.81	0.435^c^
<18.5	2 (7)	0	
18.5 to <25	16 (55)	5 (63)	
≥25	11 (38)	3 (38)	
Systemic diseases, *n*	7 (24)	3 (38)	0.655^b^
HT	5	2	0.631^b^
HL	4	2	0.591^b^
DM	2	0	
FLD	1	0	
Causative gene, *n*			
Autosomal-dominant			
*RHO*	1	0	
*TOPORS*	1	0	
Autosomal-recessive			
*EYS*	4	0	
*USH2A*	2	0	
*RP1L1*	1	0	
*PDE6B*	2	0	
X-linked	0	0	
Not determined	15	7	0.232^b^
Not tested	3	1	1^b^

*CFT*, central foveal thickness; *EZL*, ellipsoid zone length; *MD*, mean deviation; *BMI*, body mass index; *CME*, cystoid macular edema; *DM*, diabetes mellitus; *ERM*, epiretinal membrane; *FLD*, fatty liver disease; *HL*, hyperlipidemia; *HT*, hypertension; *IQR*, interquartile range; *logMAR*, logarithm of the minimal angle of resolution; *MH*, macular hole; *VA*, visual acuity; *VMTS*, vitreomacular traction syndrome.

a Data are the mean ± SD.

b Fisher’s exact test.

c Mann–Whitney *U* test.

### 3.2 Intraocular and systemic inflammatory molecules associated with RP-CME

**Figure 1:**
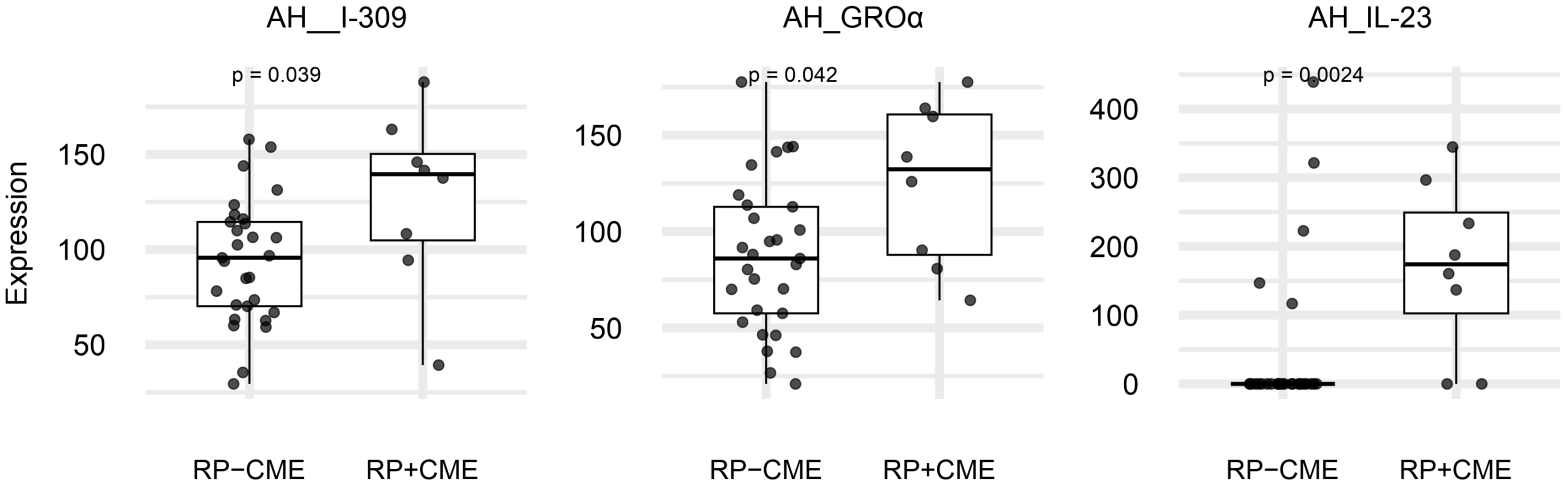
The expression levels of the significantly increased aqueous molecules in retinitis pigmentosa (RP) patients with cystoid macular edema (CME) (*n* = 8) *vs*. those without CME (*n* = 29). Box plots (median values and interquartile ranges) display the aqueous levels of IL-23, I-309, and GROα in the RP with CME eyes (*n* = 8) and the RP without CME eyes (*n* = 29). The group data were compared using the Mann–Whitney *U* test.

**Figure 2:**
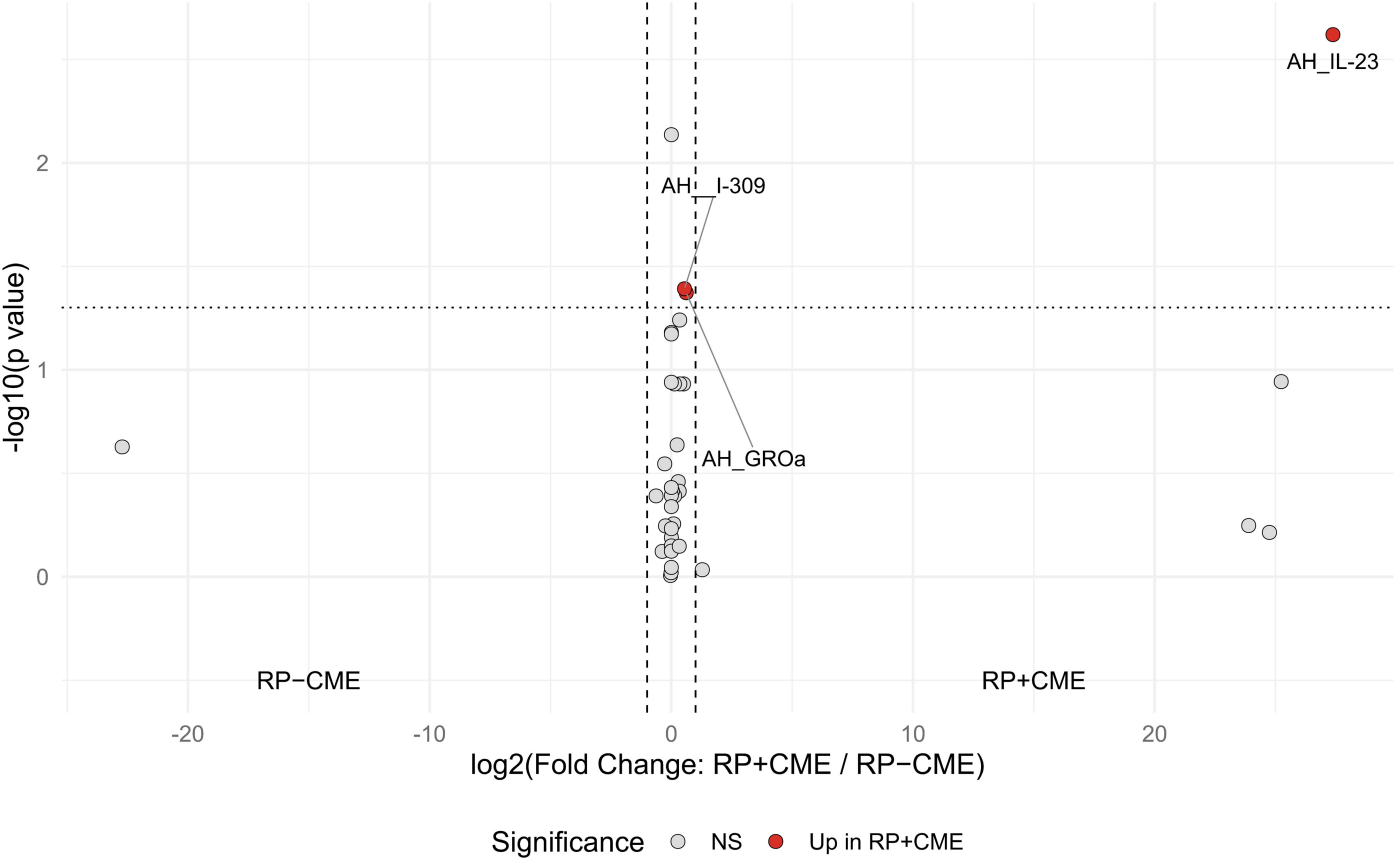
Volcano plot of the expressions of the aqueous and serum inflammatory molecules in the retinitis pigmentosa (RP) patients with cystoid macular edema (CME) *vs*. those without CME. Each *point* represents a cytokine or chemokine, with red dots indicating significant differences (Mann–Whitney *U* test: *p* < 0.05). Horizontal dotted line indicates the significance threshold (*p* = 0.05). Vertical dashed lines: |log_2_(fold change)| = 1. Analyses were performed using R statistical software.

Compared to the RP eyes without CME, those with CME displayed higher aqueous concentrations of IL-23, I-309, and GROα (IL-23: *p* = 0.002, I-309: *p* = 0.039, GROα: *p* = 0.042), as shown in **Figure 1** and **Table 2**. Among them, IL-23 showed the greatest fold increase, which is highlighted in the volcano plot in **Figure 2**. In contrast, no serum molecules were elevated in patients with CME. Furthermore, a multivariable logistic regression model including age and aqueous IL-23 confirmed that aqueous IL-23 was significantly associated with CME status (*p* = 0.02), whereas age showed no significant association (*p* = 0.06) (**Supplementary Table S1**).

**Table 2:** Differences in the aqueous and serum cytokines and chemokines between the retinitis pigmentosa (RP) patients without and with cystoid macular edema (CME).

Cytokines/Chemokines		RP without CME			RP with CME			*p*-value
		Detection rate (%)	Median	IQR	Detection rate (%)	Median	IQR	
IL-1α	AH	3	0	0–0	13	0	0–0	0.347
	Serum	14	0	0–0	13	0	0–0	0.877
IL-1β	AH	3	0	0–0	0	0	0–0	0.599
	Serum	10	0	0–0	0	0	0–0	0.35
IL-2	AH	0	0	0–0	0	0	0–0	NA
	Serum	52	6.9	0–41.0	25	0	0–12.2	0.228
IL-4	AH	0	0	0–0	0	0	0–0	1
	Serum	17	0	0–0	13	0	0–0	0.687
IL-5	AH	0	0	0–0	25	0	0–6.1	NA
	Serum	18	0	0–0	13	0	0–0	0.73
IL-6	AH	90	42.4	21.4–70.3	100	27.4	12.8–61.9	0.396
	Serum	28	0	0–7.2	13	0	0–0	0.391
IL-10	AH	0	0	0–0	0	0	0–0	1
	Serum	48	0	0–70.1	13	0	0–0	0.11
IL-12	AH	0	0	0–0	13	0	0–0	0.057
	Serum	24	0	0–2.2	13	0	0–0	0.442
IL-13	AH	0	0	0–0	0	0	0–0	1
	Serum	14	0	0–0	25	0	0–3.3	0.565
IL-15	AH	0	0	0–0	0	0	0–0	1
	Serum	17	0	0–0	13	0	0–0	0.73
IL-17	AH	10	0	0–0	0	0	0–0	0.35
	Serum	48	0	0–164.0	50	15.5	0–36.4	0.552
**IL-23**	**AH**	**18**	**0**	**0–0**	**75**	**174.0**	**34.2–280.8**	0.002*
	Serum	21	0	0–0	50	39.5	0–219.9	0.109
IFN-γ	AH	3	0	0–0	25	0	0–79.2	0.061
	Serum	48	0	0–51.0	63	28.2	0–49.4	0.597
TNFα	AH	0	0	0–0	0		0–0	NA
	Serum	83	57.2	22.7–85.6	88	71.8	23.0–115.1	0.698
TNFβ	AH	0	0	0–0	0	0	0–0	NA
	Serum	3	0	0–0	0	0	0–0	NA
Eotaxin	AH	100	21.4	18.3–24.8	100	27.1	23.2–38.5	0.055
	Serum	100	144.1	107.1–171.3	100	170.1	134.7–192.4	0.223
**GROα**	**AH**	**100**	**86.0**	**55.3–113.3**	**100**	**132.3**	**83.1–162.9**	0.042*
	Serum	100	72.7	64.8–88.2	100	88.6	68.3–109.0	0.337
**I-309**	**AH**	**100**	**95.7**	**68.6–115.3**	**100**	**139.6**	**97.9–158.9**	0.039*
	Serum	100	40.9	35.2–62.2	100	51.2	34.8–74.0	0.376
IL-8	AH	100	27.9	23.9–32.8	100	39.6	29.5–41.9	0.113
	Serum	100	38.5	15.5–144.0	100	29.6	19.6–62.6	0.74
IP-10	AH	100	270.4	199.4–384.8	100	264.1	185.2–380.8	0.971
	Serum	100	170.3	138.3–219.4	100	217.9	155.7–298.8	0.113
MCP-1	AH	100	971.2	668.9–3,885.5	100	2,361.8	616.3–3,885.5	0.909
	Serum	100	275.0	221.1–319.4	100	300.7	268.7–383.8	0.113
MCP-2	AH	86	58.6	43.8–75.6	100	64.4	50.4–94.4	0.396
	Serum	100	79.8	69.0–90.9	100	79.3	62.3–103.2	0.941
RANTES	AH	100	77.7	63.3–99.8	100	82.9	45.7–97.4	0.543
	Serum	100	4,201.2	2,850.4–5,670.4	100	3,550.6	2,651.4–5,348.3	0.555
TARC	AH	90	81.2	53.0–116.0	100	67.2	37.8–91.1	0.276
	Serum	100	215.5	155.7–247.2	100	223.1	162.0–299.1	0.376

All values are in picograms per milliliter. The *p*-values were obtained using the Mann–Whitney *U* test for the differences in the cytokines and chemokines between the RP without CME and RP with CME groups. Significant *p*-values are in bold.

*p < 0.05

*AH*, aqueous humor; *GROα*, growth-related oncogene alpha; *IL*, interleukin; *IP-10*, interferon gamma-inducible protein 10; *IQR*, interquartile range; *MCP*, monocyte chemotactic protein; *NA*, not applicable; *RANTES*, regulated activation normal T-cell expressed and secreted; *TARC*, thymus and activated-regulated chemokine.

### 3.3 Associations between peripheral leukocytes and RP with CME

**Figure 3:**
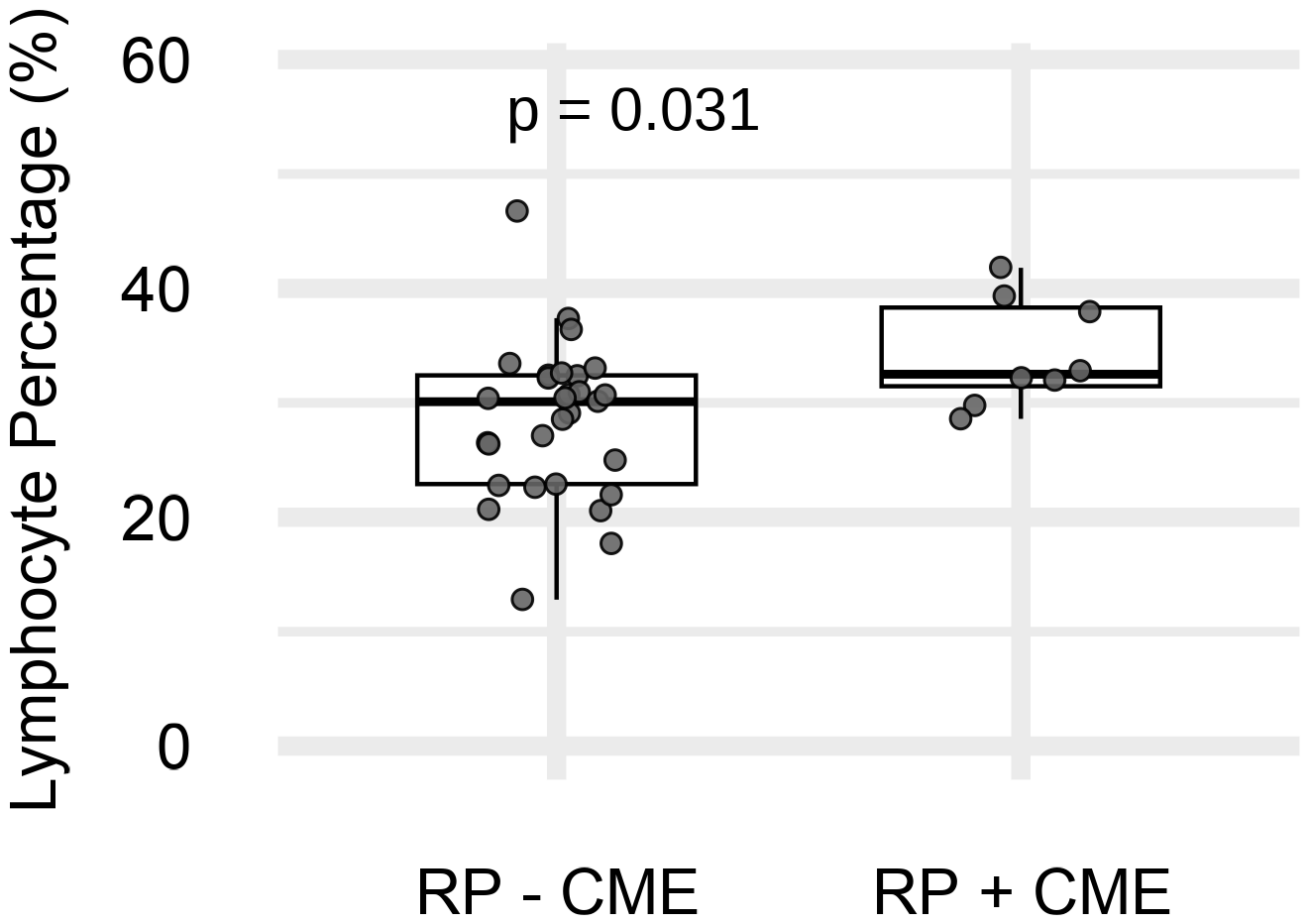
The peripheral lymphocyte percentage (%LYMPH) in retinitis pigmentosa (RP) patients with (*n* = 8) and without (*n* = 29) cystoid macular edema (CME). *p* = 0.031 (Mann–Whitney *U* test).

We next analyzed the associations between peripheral leukocytes and RP with CME (RP-CME). The peripheral blood analysis revealed a statistically higher %LYMPH in the RP-CME patients compared to patients without CME (**Figure 3**, **Table 3**).

**Table 3:** Comparison of the systemic blood parameters between the retinitis pigmentosa (RP) patients with and without cystoid macular edema (CME).

Blood parameter	RP without CME		RP with CME		*p*-value
WBC	5.5	4.6–7.3	5.1	4.3–6.3	0.373
%NEUT	59.9	55.1–65.4	55.1	49.7–59.9	0.073
**%LYMPH**	**30.1**	**22.9–32.4**	**32.5**	**30.4–39.0**	**0.031**
%MONO	5	4.2–6.3	5.2	4.8–6.8	0.502
CRP	0.04	0.03–0.1	0.05	0.03–0.07	0.677
**LNR**	**0.5**	**0.4–0.6**	**0.6**	**0.5–0.8**	**0.032**

Data are the median and interquartile range (IQR). Statistical significance was assessed using the Mann–Whitney *U* test. Significant *p*-values are in bold.

*%LYMPH*, lymphocyte percentage; %*MONO*, monocyte percentage; %*NEUT*, neutrophil percentage; *CRP*, C-reactive protein; *LNR*, lymphocyte-to-neutrophil ratio; *WBC*, white blood cell count

### 3.4 Multiple-factor analysis of the relationships between inflammatory profiles and RP-CME

Building on our research group’s previous identification of systemic–ocular inflammatory networks in RP, we performed an MFA using the same biologically defined inflammatory clusters to explore their relationships across the 37 RP patients with and without CME (10). The MFA in the present study was conducted to integrate the systemic and ocular inflammatory molecules, the CFT, and the peripheral immune parameters, aiming to determine how patterns of inflammatory molecules relate to RP-CME.

**Figure 4:**
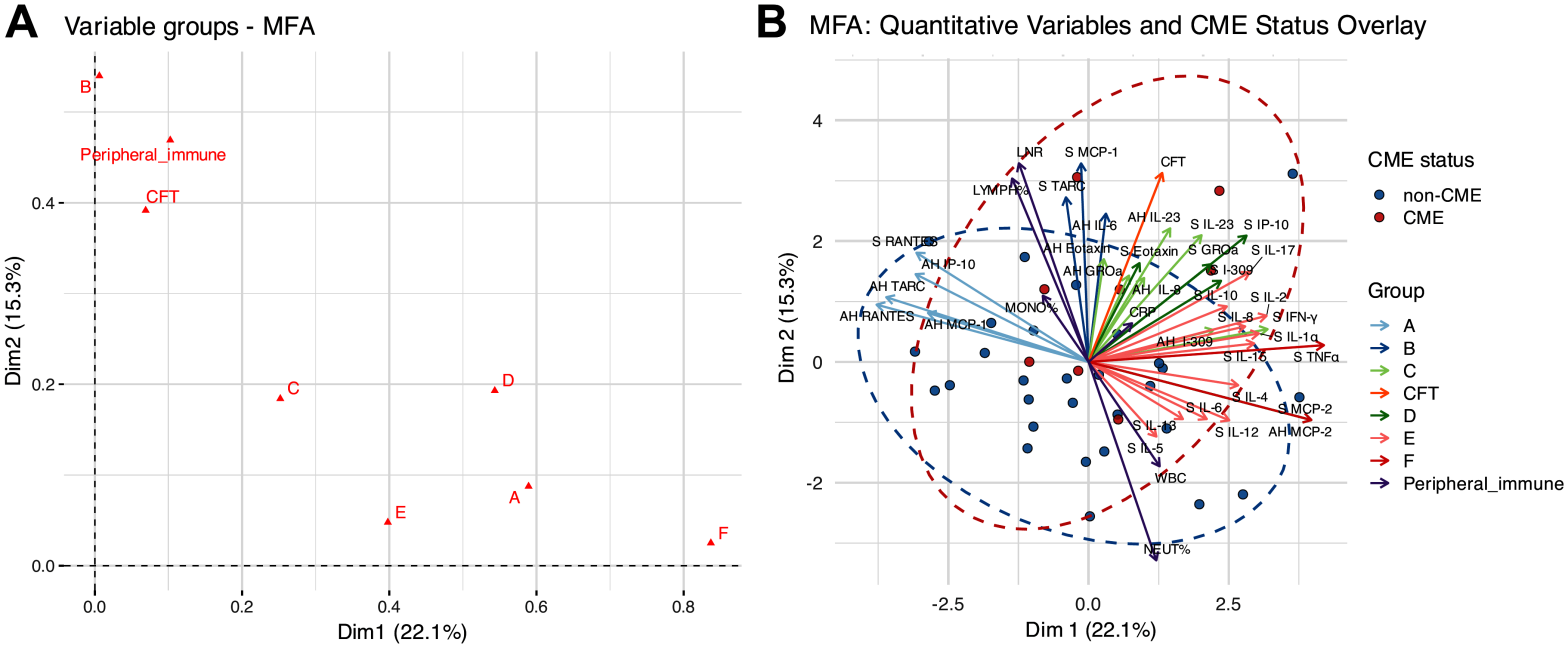
The results of the multiple-factor analysis (MFA) for the cytokines/chemokines, central foveal thickness (CFT), and peripheral immune parameters. **(A)** Global group plot. **(B)** Correlations between the quantitative variables and dimensions.

The analysis results revealed that the first two dimensions of the MFA accounted for 37.4% of the total variance, with dimension 1 (Dim1) and dimension 2 (Dim2) explaining 22.1% and 15.3% of the variability, respectively (**Figure 4A**). As shown in **Figure 4B**, the plot of the quantitative variables demonstrated that the following were closely aligned with CFT: aqueous IL-23, IL-8, eotaxin, GROα, and I-309, as well as serum IL-23 and IFN-γ from group C; aqueous IL-6, serum MCP-1, and TARC from group B; serum eotaxin, GROα, I-309, and IP-10 from group D; and the peripheral immune markers %LYMPH and LNR. These results suggest potential associations between these immune profiles and the central macular structure. In addition, these vectors were oriented in the same direction as that in the RP-CME group, indicating that these inflammatory signatures may be related to the development of CME.

In contrast, the variables from group A (aqueous RANTES, TARC, IP-10, MCP-1, and serum RANTES) and group F (aqueous MCP-2 and serum TNFα) were oriented orthogonally to the CME axis, indicating minimal associations with the patients’ CME status.

## 4 Discussion

The results of this study demonstrated that RP-CME is associated with both ocular and systemic immune dysregulation based on the following: 1) the RP patients with CME exhibited ocular upregulations of IL-23, I-309, and GROα; 2) the peripheral %LYMPH was significantly higher in patients with CME compared to those without CME; and 3) the MFA revealed potential associations between the CME status of RP patients and several IL-23-related inflammatory network parameters (i.e., aqueous IL-23, IL-8, GROα, eotaxin, I-309, and serum IL-23 and IFN-γ) and the peripheral immune markers %LYMPH and LNR.

These findings extend our previous work, which identified a systemic–ocular inflammatory interaction in RP and highlighted aqueous IL-23 as a potential molecule associated with disease progression. The present findings further demonstrated an association between higher aqueous IL-23 levels and RP-CME. Moreover, as shown by the MFA results, the IL-23-related network including aqueous IL-23, eotaxin, GROα, IL-8, and I-309, as well as serum IL-23, was spatially aligned with the CFT and the CME status of the patients. IL-23 is known to promote Th17/Tc17 polarization and induce downstream cytokines (e.g., IL-17A/F), which in turn disrupt the BRB, recruit leukocytes, and activate Müller-cell swelling pathways (14, 16, 17). These processes may collectively contribute to the formation of RP-CME. Given that inhibitors of the IL-23/IL-17 axis have already been approved for the treatment of psoriasis and other immune disorders, this pathway may serve as a potential therapeutic target for RP-CME (18, 19).

Elevated ocular levels of I-309 (also known as CCL1) and GROα (CXCL1), which are key chemokines involved in lymphocyte and neutrophil recruitment, further support the notion of active immune cell infiltration into the ocular environment (16, 20). In addition, our findings indicate that peripheral lymphocytes may be activated in patients with RP-CME, which is consistent with our previous findings of a local–systemic inflammatory interaction in patients with RP (10, 15). Given the presence of various lymphocyte subsets in the vitreous and aqueous of individuals with RP (11, 21, 22), we suspect that lymphocyte-related immune activation may be implicated in BRB dysfunction and increase the susceptibility to CME.

The RP-CME patients were significantly older than those without CME. A previous study has reported significant associations between older age, greater central macular thickness, and worse vision in RP patients with CME (23). Mechanistically, an age-related decline in BRB integrity, Müller cell vulnerability, and a dysregulated para-inflammation in the aging retina may increase the susceptibility to CME (24–26). However, in our cohort, the multivariable logistic regression model revealed that the aqueous IL-23 levels were independently associated with the CME status, whereas age did not reach statistical significance. These results suggest that an increased inflammatory activity may contribute to the development of RP-CME, potentially beyond the effect of age.

In the MFA, the distance from the origin reflects the strength of association with CFT and the IL-23-related variables. Three CME-positive cases positioned close to the origin showed trends toward younger age, better visual acuity, and thinner CFT, whereas three cases far from the origin tended to be older and with thicker CFT. This may suggest that IL-23 pathways may also be associated with greater CME severity.

No causative variants were detected in the RP-CME cases, whereas the detection rate in RP without CME (~40%) was consistent with previous reports in the Japanese population (27). Although the underlying reason remains uncertain, the limited sample size may be a contributing factor. Although subacute autoimmune retinopathy was excluded based on the clinical course and findings, chronic inflammation due to non-genetic factors may still underlie the disease in some cases (17). We are currently conducting whole-genome sequencing in genetically unsolved patients with RP and will further investigate the relationship between RP causative genes and CME.

This study has several limitations. Sample size of the RP-CME group (*n* = 8) was relatively small, which may limit the generalizability of the findings and the statistical power. Although the %LYMPH and the LNR showed statistically significant differences, the absolute differences were modest. Given that the LNR is a peripheral marker that may not fully reflect local ocular inflammatory activity, and that no concurrent increase in the serum inflammatory mediators was observed to support a systemic inflammatory explanation, their clinical significance remains uncertain. In addition, there were two RP-CME patients who had coexisting VMTS, which may have contributed to CME through mechanical or inflammation-related mechanisms (9, 28, 29). Their inclusion may introduce heterogeneity. In addition, due to the cross-sectional design of the study, causality between inflammatory activity and CME cannot be established. Moreover, although predefined inflammatory clusters from our previous research were used to enhance the biological relevance of the present findings, these groupings require validation in independent RP populations.

In conclusion, the results of this study highlight an IL-23-related pathway and peripheral immune activation as potential factors associated with the development of CME in patients with RP. Further longitudinal studies in larger cohorts are warranted to clarify these relationships and explore their clinical implications.

## Data Availability

The raw data supporting the conclusions of this article will be made available by the authors, without undue reservation.

## Supplementary material

Supplementary Table 1 Multivariable logistic regression analysis of factors associated with CME in RP patients Age Odds ratios (ORs), 95% confidence intervals (CI), and P values are shown. The model included age (per year) and aqueous IL-23 concentration (per pg/mL) as independent variables.
