# Supplementary material for: Ocular and systemic immune profiles associated with cystoid macular edema in retinitis pigmentosa
Source: Front Ophthalmol (Lausanne). 2025 Sep 5;5:1653404. doi: 10.3389/fopht.2025.1653404 (PMC12446031; doi:10.3389/fopht.2025.1653404)
Supplement: Supplementary Table 1 — Multivariable logistic regression analysis of factors associated with CME in RP patients Age Odds ratios (ORs), 95% confidence intervals (CI), and P values are shown. The model included age (per year) and aqueous IL-23 concentration (per pg/mL) as independent variables. [file Table1.docx]

**Supplement Table S1.** Multivariable logistic regression analysis of factors associated with CME in RP patients

| **Variable** | **OR (95% CI)** | **P value** |
| --- | --- | --- |
| Age | 1.10 (0.99–1.23) | 0.06 |
| Aqueous IL-23 | 1.01 (1.00–1.02) | 0.02 |

Age Odds ratios (ORs), 95% confidence intervals (CI), and P values are shown. The model included age (per year) and aqueous IL-23 concentration (per pg/mL) as independent variables.
